# Extensive inter-strain diversity among clinical isolates of *Shigella flexneri* with reference to its serotype, virulence traits and plasmid incompatibility types, a study from south India over a 6-year period

**DOI:** 10.1186/s13099-019-0314-9

**Published:** 2019-06-14

**Authors:** Ankita Das, Jharna Mandal

**Affiliations:** Department of Microbiology, JIPMER, Puducherry, India

**Keywords:** *Shigella flexneri*, Virulence, O-antigen, Serotype variation, Plasmid incompatibility typing

## Abstract

**Background:**

*Shigella* has evolved as a result of acquiring extragenetic material through horizontal gene exchange. These aid in the rapid emergence of bacterial inter-strain diversity in virulence factors and serotype variants through O-antigenic switching. Plasmid incompatibility typing of isolates is insightful in understanding local expansion of virulence plasmids, as whether virulence dissemination involves diverse plasmids or one dominant ‘epidemic’ type. The broad question underlying this study was that of how inter-strain genetic, serotype and plasmid incompatibility type variations can help understand the emergence of Shigella as a highly virulent pathogen.

**Results:**

A total of 101 confirmed isolates of *S. flexneri* were included in this study. The distribution of the subtypes were variable, type 2a (48/101, 47.5%), type 6 (15/101, 14.9%), type 1b (8/101, 7.9%), type 1 variant (7/101, 6.9%), type 3b (12/101, 11.9% 0, type 4 (6/101, 6.0%), variant Y (2/101, 1.9%) and variant X (1/101, 1%). All had the *ipaH* gene (101/101, 100%) followed by *ompA* (92/101, 91.1%), *ial* (84/101, 83.4%), *sen* (82/101, 81.2%), *virF* (84/101, 83.2%), *set1A* and *set1B* (59/101, 58.4%). Out of the total of 49 isolates that showed all the virulence related genes studied here the IncIγ plasmid was detected in all isolates studied followed by FII (33/49, 67.3%), FIIS (20/49, 40.8%). Inc K was positive in two isolates (2/49, 4%) studied. The inc groups IncI1-α, Inc T were detected in 1 isolate each and Inc L and Inc P formed part of the multireplicon in the same isolate.

**Conclusions:**

In order to estimate the burden of the disease caused by the new serotypes, it is important to have knowledge of the locally prevalent serotype. This will prove helpful in developing strategies for prevention of same especially since, the immunity in such diseases is serotype specific. Thus, the emergence of non-typable atypical serotypes of *S. flexneri* from natural infections needs to be investigated further. This study highlights the emergence of genetic variants exhibiting resistance to many antibiotics which needs to be studied for understanding the ever-changing landscape of this pathogen.

**Electronic supplementary material:**

The online version of this article (10.1186/s13099-019-0314-9) contains supplementary material, which is available to authorized users.

## Background

In early 2017, WHO (World Health Organisation) published its first ever list of antibiotic-resistant “priority pathogens”—a catalogue of 12 families of bacteria that pose the greatest threat to human health. Shigella figured in this list as a priority AMR (antimicrobial resistance) pathogen [1]. Shigella is one of the leading bacterial causes of severe and fatal childhood diarrheal disease worldwide and has become infamous due to its AMR. The adequacy of laboratory adapted reference strains to study “real world “pathogenesis of strains needs further investigation since current knowledge indicates that inter-strain variations are important to understand microbial evolution, adaptations, pathogenicity, transmission and resistance. Moreover, Shigella is constantly evolving because of acquiring extragenetic material through horizontal gene exchange in the form of plasmids, transposons, pathogenicity islands etc. thus making it imperative to bank on the knowledge of its genetic diversity and variations to have an insight about these fundamental epidemiological shifts and emergence of highly resistant pathogens.

A varied distribution of serotypes has been reported by few studies conducted worldwide, though among all the serogroups, the serogroup B of Shigella is more prevalent in the developing and less developed regions. *S. flexneri* can be divided into serotypes based on lipopolysaccharide (LPS) O-antigen structure. According to Boyd’s typing scheme, *S. flexneri* serotypes are defined by a series of type and group antigens [2–4]. Type antigens are designated by Roman numerals I to VI and are found only within one serotype (e.g., serotype la and lb share the type antigen I). Group antigens are designated by Arabic numerals 3 (4), 6, and 7 (8). These group antigens can be shared among the different serotypes. A different combination of type and group antisera results in 6 serotypes (I, II, III, IV, V, VI) and 13 subtypes (1a, 1b, 2a, 2b, 3a, 3b, 4a, 4b, 5a, 5b, 6, X, Y). Emergence of new sub serotypes is becoming a major issue, as these strains are non-typeable by conventional methods. The knowledge of a prevalent serotype in various geographic regions may assist in formulating strategies such as the development of a vaccine to prevent infection especially when the immunity to disease is serotype specific, and to understand the disease burden caused by new serotypes [5]. More recently, it has become clear that other serotypes are also epidemiologically important. A type 1c (also called 7a) was identified in Bangladesh in the late 1980s, which resulted due to seroconversion of type 1a by addition of a second glucosyl group linked to tetrasaccharide repeating unit of the O antigen. A recent study reports an acquisition of *gtrIC* gene cluster via a bacteriophage to be responsible for this seroconversion from type 1a to 1c [6, 7]. Epidemiological surveillance in China documented a new serotype of *S. flexneri* which appeared in 2001 and replaced serotype 2a in 2003 as the most prevalent serotype [8]. The new serotype was identified to be a variant of serotype X which agglutinated with the monovalent anti-IV type antiserum and the group antigen-specific monoclonal antibody MASF IV-I. It was labelled as serotype X variant (Xv). Similar studies have reported 4a variant (4Av), 4, Y variant (Yv), 7b, Z thus taking the presently known subtypes of *S. flexneri* to 23 subtypes. O-antigen modification by glycosylation or O-acetylation in *S. flexneri* is mediated by serotype-converting bacteriophages or prophages (SfI, SfIC, SfII, Sf6, SfIV, SfV and SfX) carrying the serotype conversion gene modules (*gtrABC* or *oac*) [9, 10]. These emerging atypical and novel sub-serotypes warrants more in-depth studies into evolutionary flexibility in Shigella.

Shigella possesses a diverse and unique set of genes which operate in a concerted manner through the type III secretion system to cause the disease pathogenesis. *ipaH* is the invasion plasmid antigen along with *ial* the invasion associated locus, which assists in intracellular growth by facilitating its penetration into intestinal cells [11]. These genes are effectors of the type III secretion system (T3SS). Shigella enterotoxin 1 (ShET1) encoded by *set1A* and *set1B* genes encoded within the SHI-1 pathogenicity island along with Shigella enterotoxin 2 (ShET2) encoded by the *ospD3* (*sen*) on the virulence plasmid are the two major enterotoxins secreted by T3SS. This is responsible for watery diarrhoea symptoms associated with *S. flexneri* infections [12, 13]. *VirF* is the master regulator of virulence gene expression and is a member of *AraC* family of transcription activators [14]. *OmpA*, which codes for the 35kda outer membrane protein in Shigella and have an active role in the virulence of *S. flexneri* aids in intercellular spread, protrusion formation and *IcsA* exposition. It is known to be highly conserved among gram negatives and being investigated as a promising vaccine candidate [15].

Plasmids play a central role in facilitating horizontal genetic exchange and therefore promoting the acquisition and spread of resistance genes. Concerted activities of mobile genetic elements having the ability to move within or between DNA molecules also aid in dissemination of virulence genes among highly resistant and highly virulent *S. flexneri*. Shigella strains carry a large (120- to 140-megadalton) plasmid called pINV, which contains genes necessary for the invasive behaviour of these pathogens along with the genes mediating antibiotic resistance and other survival fitness. Plasmids associated with the dissemination of virulence genes are typed using PCR based replicon typing (PBRT), which target the replicons of major plasmid families. Currently, 28 Inc groups are recognized in the Enterobacteriaceae family including six IncF and three IncI variants. Major plasmid families occurring in Enterobacteriaceae are HI2, HI1, I1-γ, X, L/M, N, FIA, FIB, FIC, W, Y, P, A/C, T, K, B/O, FII, FIII, FIV [16, 17].

Currently, data on virulence factors and serotype distribution of *S. flexneri* strains from India is limited and there is no information on the plasmid incompatibility groups in *S. flexneri*. In this study we focussed on how inter-strain variations are related to the emergence of Shigella as a highly virulent and elusive pathogen.

## Results

In the study period all together 226 isolates of Shigella were identified, of which 101 (37.7%) isolates were confirmed to be *S. flexneri*, 59 (22.0%) were *S. sonnei* followed by 8 (2.6%) *S. boydii* and 6 (2.2%) *S. dysenteriae* strains, 52 (35.45%) of the isolates were non-typable Shigella which did not agglutinate with any of the antisera tested. These isolates failed to agglutinate even after an attempt was made to remove the capsular antigen by boiling as recommended by WHO before serotyping [18].

### Subtype trends

Since, majority of our isolates were *S. flexneri*, subtyping was done further for this serogroup. Differences in subtype distribution of *S. flexneri* were seen among the isolates studied (Additional file 1: Table S1). The eight most frequently isolated *S. flexneri* serotypes was type 2a (48/101, 47.5%), type 6 (15/101, 14.9%), type 1b (8/101, 7.9%), type 1 variant (7/101, 6.9%), type 3b (12/101, 11.9%), type 4 (6/101, 6.0%), variant Y (2/101, 1.9%) and variant X (1/101, 1%) as shown in Table 1. None of the isolates were positive for type 5 subtype while, two isolates (2.0%) were non-typable. The findings of this study are based on the use of polyclonal antisera for *S. flexneri* [19]. Monoclonal antibodies diluted for slide agglutination under the advanced serological scheme could not be followed in this study due to lack of funds [20].

**Table 1:** Distribution and agglutination reactions of *S. flexneri* isolates in this study

			Antisera specific for all type- and group-factor antigens									
				Typing sera						Grouping sera		
Isolate	Serotype		B	I	II	III	IV	V	VI	3 (4)	6	7 (8)
*S. flexneri*	Type 2a	48/101	+	−	+	−	−	−	−	+	−	−
	Type 6	15/101	+	−	−	−	−	−	+	−	−	−
	Type 1b	8/101	+	+	−	−	−	−	−	−	+	−
	Type 1 variant^*^ (1v)	7/101	+	+	−	−	−	−	−	−	−	−
	Type 3b	12/101	+	−	−	+	−	−	−	−	+	−
	Type 4	6/101	+	−	−	−	+	−	−	−	−	−
	Variant X	1/101	+	−	−	−	−	−	−	−	−	+
	Variant Y	2/101	+	−	−	−	−	−	−	+	−	−
	Nontypable *S. flexneri*	2/101	+	−	−	−	−	−	−	−	−	−

*Identified in this study only

### Virulence gene profile

Virulence genes showed variability in presence across all serotypes of *S. flexneri*. The detection of *ipaH* (invasion plasmid antigen H) genes showed that it was positive in all the isolates (100%). The *ial* (invasion associated locus) is located only on the plasmid and as a result is prone to lose or deletion so was positive in 84/101 (83.4%) of our isolates. Shigella enterotoxin 1 (ShET1) is a 55 KD protein, encoded by chromosomal genes *set1A* (known previously to be reported only in *S. flexneri* strains, but studies from our lab [21] and few other studies have found it in *S. sonnei*) and *set1B* was positive in 59/101 (58.4%). *Sen* coding for Shigella enterotoxin 2 (ShET2), was positive in 82/101 (81.2%). *VirF*, the virulence regulon transcriptional activator located both on the large virulence plasmid and the chromosome was detected in 84/101 (83.2%) isolates. *OmpA*, which codes for the outer membrane protein in Shigella is known to have a role in the molecular pathogenesis of shigellosis. They are being targeted for their role as potential biomarkers. It is known to be highly conserved among gram-negative bacteria, but it was not seen to be conserved and was seen in 92/101 (91.1%) of our isolates only. Also 48.5% (49/101) isolates were positive for all the virulence genes tested and these strains were taken in for plasmid incompatibility.

### Replicon typing

PBRT-KIT identified replicons in 100% of the analysed isolates under study. IncIγ incompatibility group which belongs to the I-complex plasmid family was detected in 100% of the isolates studied. All the replicons were found to exist only in a multi-replicon state among the isolates, except two isolates which had the IncIγ plasmid only. Among the F-family of plasmid which is referred to as the fertility factor (also known as F factor or sex factor), inc group FII was detected in 67.3% (33/49) isolates followed by FIIS in 40.8% (20/49) of the isolates. Inc K was positive in two isolates studied. The inc groups IncI1-α, Inc T were detected in 1 isolate each and Inc L and Inc P formed part of the multireplicon in the same isolate (Additional file 1: Table S2).

## Discussion

In reports published from WHO, *S. flexneri* is reported as the most frequently isolated species worldwide, accounting for majority of the cases in the developing countries, while *S. sonnei* is more common in low- and middle-income countries. In comparison, *S. dysenteriae* especially *S. dysenteriae* type 1, has become largely resistant to antibiotics and has historically caused epidemics of dysentery and is particularly common in closed populations such as refugee camps and has acquired the infanity of being a stubborn killer of refugees [22]. *S. boydii*, accounts for 6 percent or less of Shigella cases in less-developed countries. This study too demonstrated similar distribution of Shigella in our geographical region with *S. flexneri* being the most dominant followed by *S. sonnei* and then similar distribution of *S. boydii* and *S. dysenteriae.* Various studies have concluded that *S. sonnei* prevalence increases with economic development and *S. sonnei* has a greater ability to develop resistance to broad-spectrum antimicrobials. *S. sonnei* has a competitive edge over *S. flexneri* as it can accept and maintain with more stability horizontally transferred DNA which might be contributing to its gradual increasing dominance [23, 24].

Isolation of uncommon serotypes and sub-serotypes of *Shigella* spp. particularly of *S. flexneri* is not a rare event. In this study we demonstrated the predominance of *S. flexneri* 2a which is in concordance with all the studies done so far [5, 25, 26]. Further, serotype 6 was the most dominant. Heterogeneity in distribution of other serotypes was noticed among isolates in this study as has been elaborated in Additional file 2: Table S3 [5,10, 26–29]. The mixture of other serotypes identified in this study is disparate, thus showing that there is no homogenous distribution of serotypes globally as shown in Table 2. It is thus important to not only pay close attention to the newly emerged serotypes but also to keep a record of predominant subtypes circulating in the geographical region. Based on recent Global Enteric Multicenter Study (GEMS) finding a heat killed multi-serotype Shigella (HKMS) vaccine was developed at National Institute of Cholera and Enteric Diseases (NICED), Kolkata combining the following six Shigella strains: *S. dysenteriae* 1 (NT4907stx), *S. flexneri* 2a (B294), *S. flexneri* 3a (C519), *S. flexneri* 6 (C347), *S. sonnei* (IDH00968) and *S. boydii* 4 (BCH612) [30, 31]. The present study deviates and projects a different picture of the dominant serotypes in the southern part of the country with type 2a, 6 and 3b being the dominant subtypes in our study. This might limit the HKMS vaccine application in this part of the country. Based on agglutination reaction some rare atypical isolates which was labelled as type I variant (1v) was also detected in this study which has not been reported earlier. This was followed by few type 4 isolates.

**Table 2:** Frequency of the serotype distribution of *S. flexneri* subtypes

National and International studies	*S. flexneri* subtypes identified in order of dominance
Present study, India (n = 101)	2a > 6 > 3b > 1b > 1v > 4
Kolkata, 2010, India (n = 154)	2a > 3a
Kolkata, 2014, India (n = 47)	2a > 3a > 6 > 4a > 7a/1c
Bay of Bengal Islands, India 2014 (n = 55)	2a > 4a > 3b = X > 3a = 1a = 6
China, 2013 (n = 4295)	2a > 4c/Xv > X > 4a = 1a > 2b > Y > 6 > 4b > 5a = 3a > 1b
China, 2017 (n = 545)	2a > 2b > 1a > 1b > X > 4c/Xv > Y > 6 > 4a > 3b
China, 2018 (n = 365)	2a > 1b > 2b > 6 > X > 1a > Y > 4 s/Z > 4a = 3a > 2c > 7a/1c = 3b
Bangladesh, 2014 (n = 415)	2a > 2b > 3a > 6 > 1b > 4a > 7a/1c > Y > 5b > 1a > 3b
Gems, 2014, 7 countries (n = 745)	2a > 6 > 2b > 3a > 1b > 4a > 7a/1c > X > Y>1a = 5b > 3b
Bangladesh, 2018 (n = 3569)	2a > 3a > 2b > 6>1b > 7a/1c > 4 s/Z > Y>1a > 3b = X > 4a = 5a > 4b > 5b

The burden of virulence genes among *S. flexneri* isolates in the present study showed variable distribution considering the high genetic plasticity of *S. flexneri* known due to impact of horizontal gene transfer (HGT) shaping bacterial genome. Isolates belonging to a specific *Escherichia coli* pathotype may display a known combination of virulence which can be used to identify the pathotype [32]. Similarly *ipaH* is known to be used as a marker to identify Shigella which has been confirmed by previous studies [26]. A dense distribution of most of the virulence determinants was noted among isolates with all the genes studied, showing more than 50% prevalence. The invasive genes *ipaH* was positive for all (100%) the isolates which agree with various other studies done so far. The reason might be due to its presence in both the plasmid and chromosome thus it can be detected even if the plasmid is lost. The *ial* gene is responsible for the invasiveness and is located on the inv plasmid only thus is prone to deletion. The present study found *ial* positive in 83.4% isolates which is in concordance with reports from Iran by Hossein et al. (80.4%) [33], Argentina by Casabonne et al. (83%) [34] and higher than that reported from China by Fan et al. (47%) [26].

*Set1a* and *set1b* coding for the enterotoxin Shet1 is known to be responsible for electrolyte and water transport in the small intestine causing dehydration. This gene was positive among 58.4% of our isolates and the type1v and variant Y showed absence of these genes may be suggesting that this serotype has a low ability to cause dehydration. ShET2, the other enterotoxin is encoded by *sen* gene located on the large virulence plasmid like *set1a* and *set1b*. This is the major enterotoxin among isolates (81.2%) in the present study. It may be thus predicted that *sen* is the major player to cause the symptoms of electrolyte imbalance and water loss causing dehydration among cases of shigellosis in our region. Like a study published recently by Fan et al. [26] in which type 1b showed a poor association with *sen*, *set1a* and *set1b* hinting that may be this serotype has a low ability to cause dehydration as shown in Table 3.

**Table 3:** Distribution of virulence genes among *S. flexneri* subtypes

	*ipaH*	*sen*	*ial*	*set1a*	*set1B*	*virF*	*ompA*
Type 2a	100% (48/48)	89.6% (43/48)	89.6% (43/48)	91.7% (44/48)	91.7% (44/48)	89.6% (43/48)	93.7% (45/48)
Type 6	100% (15/15)	80% (12/15)	86.7% (13/15)	20% (3/15)	20% (3/15)	93.3% (14/15)	93.3% (14/15)
Type 1b	100% (8/8)	25% (2/8)	50% (4/8)	25% (2/8)	25% (2/8)	37.5% (3/8)	87.5% (7/8)
Type 1 variant^*^ (1v)	100% (7/7)	71.4% (5/7)	71.4% (5/7)	0	0	71.4% (5/7)	85.7% (6/7)
Type 3b	100% (12/12)	83.3% (10/12)	75% (9/12)	33.3% (4/12)	33.3% (4/12)	83.3% (10/12)	75% (9/12)
Type 4	100% (6/6)	66.7% (4/6)	66.7% (4/6)	66.7% (4/6)	66.7% (4/6)	66.7% (6/6)	66.7% (6/6)
Variant X	100% (1/1)	100% (1/1)	100% (1/1)	100% (1/1)	100% (1/1)	100% (1/1)	100% (1/1)
Variant Y	100% (2/2)	100% (2/2)	100% (2/2)	0	0	50% (1/2)	50% (1/2)
Nontypable *S. flexneri*	100% (2/2)	50% (1/2)	50% (1/2)	50% (1/2)	50% (1/2)	0	100% (2/2)
TOTAL	100% (101/101)	83.4% (84/101)	81.2% (82/101)	58.4% (59/101)	58.4% (59/101)	83.4% (84/101)	91.1% (92/101)

*Identified in this study only

*VirF*, which is the master activator of virulence genes of Shigella was found to be present in 83.2% of our isolates which is higher than that has been reported in another earlier study from China (64.8%) [26]. *VirF* is encoded on the primary pathogenicity island carried by the large virulence plasmid, pINV, which is also prone to deletion. This may be the reason for the reduced frequency of *VirF* among the isolates. A recent study suggested the use of regulatory genes like *VirF* as a target for anti-virulence therapies [35]. However with this reduced frequency of prevalence it does not qualify to be a good target for therapy.

*OmpA* which is the outer membrane protein is known to be responsible for host immune responses and as targets for drug therapy. *S. flexneri* 2a *OmpA* is cross-reactive, conserved and surface exposed as per recent reports [36], but this study identified three isolates of *S. flexneri* 2a which were negative for this gene thus limiting its use as a potential vaccine candidate, though further analysis needs to be carried out to ascertain the basis of this. Current study is the first report to investigate prevalence of *ompA* among *S. flexneri* isolates.

All the virulence factors mentioned above are majorly situated in large virulent plasmids which showed diversity in Incompatibility groups they belong to. The I-complex plasmid family includes incompatibility groups IncI1, IncIγ, IncB, IncZ and IncK. In recent years, plasmids of the IncI1 and IncIγ families have been recognized in clinically relevant bacteria from human, animal and environmental sources and are majorly reported to be associated with several ESBL and AmpC producing Enterobacteriaceae [37]. IncIγ being the most dominant plasmid replicon group in our study harbouring the entire panel of virulence genotype, might suggest this replicon group to be playing a role not only as confirmed vehicle of transmission of extended spectrum beta-lactamase and plasmid *AmpC* genes but also of virulence genes in *S. flexneri*. The next most dominant incompatibility group among the isolates studied was the inc group FII which have recently been reported to be associated with fosfomycin resistance gene *fosA3* among *E. coli* Isolates from farmyard animals like cows, pigs and poultry [38]. Another study reported this conjugative plasmid circulating among animal *E. coli* isolates to be associated with transmission of the *blaNDM*-*1* gene for carbapenem resistance [39], though we did not document either fosfomycin or carbapenem resistance. The incompatibility groups IncIγ, FII, and FIIS are predicted to be associated with increased virulence among the isolates. The inc groups IncI1-α, Inc T, Inc L and Inc P which were detected in minimum number of the isolates studied are predicted to be least associated with the ability of virulence dissemination. In a study to investigate the replicon types in *S. sonnei* from Thimphu, Bhutan showed that all isolates carried ColE plasmid containing genes that code for colicin protein (bacteriocin protein) that can kill other bacteria and replicate in absence of de novo protein synthesis. Furthermore, ColE plasmids co-resided with B/O or I1 in several *S. sonnei* isolates [16]. Whole genome sequencing can be helpful to supplement PBRT results, if the evaluation of complete replicon content beside the 28 chosen replicons along with its association with virulence markers is to be studied.

The findings of the present study highlight the importance of continuous monitoring of emerging isolates. However, accurate identification of these isolates requires specific antisera as well as molecular typing tools, which remain beyond the capability of most clinical diagnostic laboratories in developing countries.

## Conclusion

Shigella travels unimpeded from the mouth to the colon, where they unleash powerful machinery to trigger debilitating diarrhoea. Each Shigella group and subgroup use various combinations of virulence factors to exert their pathogenesis on the host and consequently deserve specific in-depth comprehensive analysis. Another important feature was the emergence of non-typeable Shigella which will be studied and characterised further in future studies. The diversity in distribution of virulence genes along with the incompatibility groups dominant among the isolates, is suggestive of major HGT events contributing to the plasticity in the genome of the pathogen. The present study contributes in profiling the distribution of various serotypes, virulence traits and replicon typing of *S. flexneri* strains which is relevant to keep a note on the new emerging variants and the pattern of isolates present in our part of the country, as there are still many unanswered questions relating this infamous and elusive bacterial pathogen.

## Methods

### Bacterial isolates and settings

Isolates of Shigella obtained from diarrhoeal stool samples received in the Department of Microbiology, JIPMER from 2012 till 2017 have been included in the study. This was a descriptive study which was approved by the Institute Ethics Committee (Human studies) at Jawaharlal Institute of Postgraduate Medical Education and Research (JIPMER), Pondicherry (Approval No. JIP/IEC/2015/15/742). The isolates were confirmed biochemically and using serotyping protocols as mentioned elsewhere [18, 19]. Sero-grouping and serotyping was done by slide agglutination with serogroup specific antisera (Denka Seiken, Tokyo, Japan). Negative controls were performed using 0·9% NaCl instead of antibody. Since serogroup B was found to be the most predominant circulating serogroup with notable variations, this study focuses on the same. Occasionally, the presence of capsular antigens may prevent some isolates of *Shigella* spp. from reacting with polyvalent antisera. To serotype such isolated the bacterial suspension were heated at 100 °C in water bath for 45 min and procedure for serotyping was repeated as mentioned above [18].

### Virulence genes profiling

Total DNA was isolated for further molecular analysis using the Kit extraction protocol for genomic DNA using the manufacturer’s protocol with the Qiagen DNA mini kit. Polymerase chain reaction was carried out for virulence profiling of isolates for investigating the array of chromosomal- and plasmid-encoded virulence genes virulence genes as follows: invasion plasmid antigen H gene (*ipaH*), invasion antigen loci (*ial*), Shigella enterotoxin 1 (ShET-1) (*set1A&set1B*), Shigella enterotoxin 2 (ShET-2) (*sen*), virulence regulator of transcription (*virF*), outer membrane protein (*ompA*). The primer details are as described in Table 4.

**Table 4:** List of primers used in the study

Gene target	Primer sequence (5′-3′)	Gene location	Amplicon size in base pair (bp)	Reference
*ipaH*	F:TGGAAAAACTCAGTGCCTCT	Plasmid and chromosome	423	[40]
	R:CCAGTCCGTAAATTCATTCT			
*ial*	F:CTGGATGGTATGGTGAGG	Plasmid	320	[40]
	R:GGAGGCCAACAATTATTTCC			
*set1A*	F:TCACGCTACCATCAAAGA	SHI-1 pathogenicity island	309	[40]
	R:TATCCCCCTTTGGTGGTA			
*set1B*	F:GTGAACCTGCTGCCGATATC	SHI-1 pathogenicity island	147	[40]
	R:ATTTGTGGATAAAAATGACG			
*sen*	F:ATGTGCCTGCTATTATTTAT	Plasmid	799	[40]
	R:CATAATAATAAGCGGTCGC			
*virF*	F:CAATGACGGTTAGCTCAGGCA	Plasmid	450	This study
	R: AAAGACGCCATCTCTTCTCGAT			
*ompA*	F:GCAGGCATTGCTGGGTAA	Plasmid	1319	[36]
	R:ACACTTGTAAGTTTTCAACTACG			

A representative amplicon for each gene detected was sequenced to validate that these primers amplified the target genes. The PCR positive amplicon was sequenced on both DNA strands with ABI 3730XL sequencer. The Contig sequences generated were computationally analysed by assembling and comparing using the Basic Local Alignment Search Tool (BLAST) of the National centre for Biotechnology Information (NCBI) alignment of the nucleotide sequences along with translated protein sequences (using Expasy server).

### Multiplex PCR for plasmid replicon typing

A total of 49 isolates of *S. flexneri* that were positive for all the virulence genes tested were examined for their plasmid replicon profile. Plasmids may contain more than one replicon (multireplicon plasmids). PBRT kit i.e. the PCR based replicon typing kit (Diatheva, Italy) for molecular typing of plasmid in Enterobacteriaceae was used [41]. This screening involved eight multiplex reactions which potentially identify 25 replicons representative of the major ones known to be prevalent amongst Enterobacteriaceae. An assessment of bacterial plasmid content was obtained using the total genomic DNA purified from the isolates, 10 μl of amplicons then was run on a 2.5% agarose gel containing ethidium bromide. Positive controls included in the kit was included in every run for every reaction.

### Statistical analysis

All categorical data has been mentioned as percentages.

## Additional files

**Additional file 1: Table S1.** The yearly distribution of Subtype trend over the study period. **Table S2.** Incompatibility profile, virulence profile and subtypes of isolates selected for plasmid incompatibility typing.

**Additional file 2: Table S3.** Serotype distribution of *S. flexneri* subtypes in comparison to other studies.

## Data Availability

Data and material will be made available upon reasonable request to the corresponding author.

## Abbreviations

WHO: World Health Organisation
GEMS: Global Enteric Multicenter Study
NICED: National Institute of Cholera and Enteric Diseases
HGT: horizontal gene transfer
