# Supplementary material for: Extensive inter-strain diversity among clinical isolates of Shigella flexneri with reference to its serotype, virulence traits and plasmid incompatibility types, a study from south India over a 6-year period
Source: Gut Pathog. 2019 Jun 14;11:33. doi: 10.1186/s13099-019-0314-9 (PMC6567616; doi:10.1186/s13099-019-0314-9)
Supplement: Supplementary file 1 — Additional file 1: Table S1. The yearly distribution of Subtype trend over the study period. Table S2. Incompatibility profile, virulence profile and subtypes of isolates selected for plasmid incompatibility typing. [file 13099_2019_314_MOESM1_ESM.docx]

**Table S1.The yearly distribution of Subtype trend over the study period**

|  | **2a** | **4** | **1b** | **3b** | **6** | **1v** | **Nontypable *S.flexneri*** | **Variant X** | **Variant Y** | **Total** |
| --- | --- | --- | --- | --- | --- | --- | --- | --- | --- | --- |
| **2012** | 6 | 1 | 2 | 1 | 1 | 2 | 0 | 0 | 0 | 13 |
| **2013** | 4 | 1 | 1 | 2 | 2 | 1 | 0 | 0 | 0 | 11 |
| **2014** | 3 | 2 | 2 | 3 | 3 | 0 | 0 | 0 | 0 | 13 |
| **2015** | 12 | 1 | 0 | 1 | 6 | 3 | 2 | 1 | 0 | 26 |
| **2016** | 15 | 0 | 2 | 4 | 2 | 1 | 0 | 0 | 2 | 26 |
| **2017** | 8 | 1 | 1 | 1 | 1 | 0 | 0 | 0 | 0 | 12 |

**Table S2. Incompability profile, virulence profile and subtypes of isolates selected for plasmid incompatibility typing**

| **Sl. No** | **Isolate number** | **Subtype** | **Virulence profile** | | | | | | | **Inc types** |
| --- | --- | --- | --- | --- | --- | --- | --- | --- | --- | --- |
|  |  |  | *ipaH* | *sen* | *ial* | *shet1A* | *shet1B* | *virF* | *ompA* |  |
|  | S452/12 | 4 | P | P | P | P | P | P | P | I1y;FII |
|  | S559/12 | 2a | P | P | P | P | P | P | P | I1y;FII |
|  | S126/12 | 2a | P | P | P | P | P | P | P | I1y;FII |
|  | S732/13 | 2a | P | P | P | P | P | P | P | I1y;FII |
|  | S762/13 | 3b | P | P | P | P | P | P | P | I1y;FII |
|  | S947/13 | 4 | P | P | P | P | P | P | P | I1y;FII |
|  | S55/14 | 2a | P | P | P | P | P | P | P | I1y;FII |
|  | S64/14 | 2a | P | P | P | P | P | P | P | I1y;K;FII |
|  | S103/14 | 1b | P | P | P | P | P | P | P | I1Y;FII |
|  | S239/14 | 4 | P | P | P | P | P | P | P | I1Y,FII |
|  | S240/14 | 3b | P | P | P | P | P | P | P | I1Y,FII |
|  | S1043/14 | 6 | P | P | P | P | P | P | P | I1Y,FII |
|  | S1054/14 | 2a | P | P | P | P | P | P | P | I1Y,FII |
|  | S143/15 | 2a | P | P | P | P | P | P | P | I1Y;FII |
|  | S232/15 | 2a | P | P | P | P | P | P | P | I1Y |
|  | S156/15 | 2a | P | P | P | P | P | P | P | I1Y;FII |
|  | S886/15 | 2a | P | P | P | P | P | P | P | I1Y |
|  | S968/15 | 2a | P | P | P | P | P | P | P | I1Y;FII |
|  | S978/15 | 2a | P | P | P | P | P | P | P | I1Y;FII |
|  | S1023/15 | 2a | P | P | P | P | P | P | P | L;P;I1Y;FIIS |
|  | S1061/15 | 2a | P | P | P | P | P | P | P | I1Y;FII |
|  | S55/15 | 2a | P | P | P | P | P | P | P | I1a, I1Y;FII |
|  | S973/15 | 2a | P | P | P | P | P | P | P | I1Y,FII |
|  | S67/15 | VARIANT X | P | P | P | P | P | P | P | I1Y;FII |
|  | S847/15 | 6 | P | P | P | P | P | P | P | I1Y;FII;T |
|  | S26/16 | 2a | P | P | P | P | P | P | P | I1Y;FIIS;FII |
|  | S50/16 | 2a | P | P | P | P | P | P | P | I1Y;FII |
|  | S217/16 | 2a | P | P | P | P | P | P | P | FIA,I1Y,FIIS,FII |
|  | S223/16 | 2a | P | P | P | P | P | P | P | FIA,I1Y,FIIS,FII |
|  | S225/16 | 2a | P | P | P | P | P | P | P | I1Y,K,FII |
|  | S137/16 | 2a | P | P | P | P | P | P | P | I1Y,FII |
|  | S24/16 | 3b | P | P | P | P | P | P | P | I1Y |
|  | S341/16 | 2a | P | P | P | P | P | P | P | I1Y,FIIS,FII |
|  | S954/16 | 2a | P | P | P | P | P | P | P | I1Y,FIIS,FII |
|  | S978/16 | 2a | P | P | P | P | P | P | P | I1Y,FII |
|  | S1091/16 | 2a | P | P | P | P | P | P | P | I1Y,FII |
|  | S597/16 | 2a | P | P | P | P | P | P | P | I1Y,FII |
|  | S1223/16 | 2a | P | P | P | P | P | P | P | I1Y |
|  | S290/17 | 2a | P | P | P | P | P | P | P | I1Y,FII |
|  | S223/17 | 2a | P | P | P | P | P | P | P | I1Y,FII |
|  | S510/17 | 2a | P | P | P | P | P | P | P | I1Y,FII |
|  | S660/17 | 3b | P | P | P | P | P | P | P | I1Y,FII |
|  | S664/17 | 2a | P | P | P | P | P | P | P | I1Y,FII |
|  | S908/17 | 1b | P | P | P | P | P | P | P | I1Y,FII |
|  | S984/17 | 2a | P | P | P | P | P | P | P | I1Y,FII |
|  | S1090/17 | 2a | P | P | P | P | P | P | P | I1Y,FII |
|  | S1108/17 | 2a | P | P | P | P | P | P | P | I1Y,FII |
|  | S1290/17 | 2a | P | P | P | P | P | P | P | I1Y,FII |
|  | S2488/17 | 4 | P | P | P | P | P | P | P | I1Y,FII |
