# Supplementary material for: Extensive inter-strain diversity among clinical isolates of Shigella flexneri with reference to its serotype, virulence traits and plasmid incompatibility types, a study from south India over a 6-year period
Source: Gut Pathog. 2019 Jun 14;11:33. doi: 10.1186/s13099-019-0314-9 (PMC6567616; doi:10.1186/s13099-019-0314-9)
Supplement: Supplementary file 2 — Additional file 2: Table S3. Serotype distribution of S. flexneri subtypes in comparison to other studies. [file 13099_2019_314_MOESM2_ESM.docx]

**Table S3. Serotype distribution of *S.flexneri* subtypes in comparison to other studies**

|  | **PRESENT STUDY,INDIA**  **(n=101)** | **KOLKATA, INDIA,2010[27]** | **KOLKATA, INDIA, 2014[28]** | **BAY OF BENGAL ISLANDS,**  **INDIA 2014[25]** | **CHINA,2013 [5]** | **CHINA,2017 [26]** | **CHINA,2018 [29]** | **BANGLADESH, 2014[28]** | **DATA FROM GEMS,**  **(7 countries) 2014[28]** | **BANGLADESH,2018[10]** |
| --- | --- | --- | --- | --- | --- | --- | --- | --- | --- | --- |
|  | N=101 | N=154 | N=47 | N=55 | N=  4295 | N=  545 | N=  365 | N=  415 | N=745 | N=3569 |
| **Serotype 1a** | 0 | 0 | 0 | 1.8 | 2.9 | 15.6 | 3.3 | 0.3 | 0.3 | 0.4 |
| **Serotype 1b** | 7.9 | 0 | 1.1 | 0 | 0.2 | 11.7 | 12.9 | 4.9 | 7.5 | 7.6 |
| **Serotype 1 variant** ^*^ | 6.9^*^ | 0 | 0 | 0 | 0 | 0 | 0 | 0 | 0 | 0 |
| **Serotype 2a** | 47.5 | 51 | 26.4 | 80 | 53.2 | 40.9 | 36.7 | 20.8 | 20.2 | 44.9 |
| **Serotype 2b** | 0 | 0 | 0 | 0 | 2.3 | 19.4 | 12.6 | 18.2 | 10.9 | 11.3 |
| **Serotype 2c** | 0 | 0 | 0 | 0 | 0 | 0 | 1.1 | 0 | 0 | 0 |
| **Serotype 3a** | 0 | 28.7 | 12.1 | 1.8 | 0.5 | 0 | 1.4 | 9.7 | 9.4 | 16.5 |
| **Serotype 3b** | 11.9 | 0 | 0 | 3.6 | 0.05 | 0.4 | 0.8 | 0.2 | 0.1 | 0.2 |
| **Serotype 4a** | 0 | 0 | 4.4 | 7.3 | 2.9 | 0.4 | 1.4 | 2.3 | 2.9 | 0.1 |
| **Serotype 4b** | 0 | 0 | 0 | 0 | 0.8 | 0 | 0.5 | 0 | 0 | 0.05 |
| **Serotype 4c/Serotype Xv[8]** | 0 | 0 | 0 | 0 | 3.2 | 3.3 | 9.9 | 0 | 0 | 0 |
| **Serotype 4** | 6 | 0 | 0 | 0 | 0 | 0 | 0 | 0 | 0 | 0 |
| **Serotype 4s/Type Z** | 0 | 0 | 0 | 0 | 0 | 0 | 1.9 | 0 | 0 | 2.7 |
| **Serotype 5a** | 0 | 0 | 5 | 0 | 0.5 | 0 | 0 | 0 | 0 | 0.1 |
| **Serotype 5b** | 0 | 0 | 0 | 0 | 0 | 0 | 0 | 0.5 | 0.3 | 0.02 |
| **Serotype 6** | 14.9 | 0 | 5.5 | 1.8 | 0.9 | 0.9 | 4.7 | 8.9 | 11 | 8.3 |
| **Serotype7a/**  **serotype 1c**  **[10]** | 0 | 0 | 2.2 | 0 | 0 | 0 | 0.8 | 1.6 | 2 | 6.9 |
| **Serotype 7b** | 0 | 0 | 0 | 0 | 0 | 0 | 0 | 0 | 0 | 0 |
| **Variant Y** | 2 | 0 | 0 | 0 | 1.9 | 1.3 | 2.2 | 0.7 | 0.4 | 1.5 |
| **Variant X** | 1 | 0 | 0 | 3.6 | 3 | 5.5 | 4.1 | 0 | 1 | 0.2 |
| **Nontypable*S.flexneri*** | 2 | 13 | 0 | 0 | 0 | 0 | 5.8 | 0 | 0 | 0 |

Note: values in percentages ; *=identified in this study only
